# Why nutraceuticals do not prevent or treat Alzheimer's disease

**DOI:** 10.1186/1475-2891-4-14

**Published:** 2005-04-12

**Authors:** Anna EO Fisher, Declan P Naughton

**Affiliations:** 1 School of Pharmacy and Biomolecular Sciences, University of Brighton, Cockcroft Building, Moulsecoomb, Brighton, UK

## Abstract

A great deal of research has pointed to deleterious roles of metal ions in the development of Alzheimer's disease. These include: i) the precipitation and aggregation of amyloid β (Aβ) peptides to form senile plaques and neurofibrillary tangles, and/or ii) the augmentation of oxidative stress by metal ion mediated production and activation of hydrogen peroxide. The growing trend in nutraceutical intake is in part a result of the belief that they postpone the development of dementias such as Alzheimer's disease. However, pathogenic events centred on metal ions are expected to be aggravated by frequent nutraceutical intake. Novel therapeutic approaches centred on chelators with specificity for copper and iron ions should be fully explored.

## Introduction

Dementia currently affects over 750,000 people in the UK, with Alzheimer's disease (AD) accounting for 55 % of those cases. As a result of increasing life expectancy, the number of people predicted to suffer from dementia is expected to rise to over 1.8 million by 2050. Currently there are no known cures for AD. Existing treatments at best only delay the progression of disease and accurate diagnosis can only be made with certainty at autopsy. Hence, there is an urgent need for new therapeutic approaches and for reliable and noninvasive methods to aid in the early diagnosis of AD and to give an indication of disease progression. Advanced AD is evident from the apparent progressive decline in cognitive function as a result of loss of neurons. These are accompanied by pathological changes including extracellular amyloid β plaques and intracellular changes called neurofibrillary tangles, which are abnormal deposits of hyperphosphorylated tau proteins [1].

Recent reports ascribe a major role for the metal ions, iron, copper and zinc in AD. Specifically the redox active metal ions Fe^2+ ^and Cu^+ ^can generate the highly reactive and oxidizing species, the hydroxyl radical via the Fenton reaction. This species is thought to account for a large proportion of oxidative modifications that are seen in AD brains including lipid peroxidation, nucleic acid oxidation and protein carbonylation. Also metal ions have been shown to be central to Aβ pathogenicity.

The Aβ peptide is a low molecular weight protein consisting of 39–43 amino acids. It has two high affinity binding sites, one specific for copper and one specific for zinc. In the Aβ1–42 peptide (the predominant Aβ species found in amyloid plaques in AD brains) the high affinity binding site binds Cu^2+ ^were estimated at log K = 17.2 and 16.3 at pH 7.4 and pH 6.6 respectively, and the low affinity binding site at log K = 8.3 and 7.5 at pH 7.4 and 6.6 respectively [2]. At physiological pH values, soluble Aβ is precipitated by Zn^2+ ^*in vitro*. However, at pH levels reflecting physiological acidosis, which is characteristic of AD, *in vitro *precipitation of Aβ is induced by cupric and ferric ions. Cupric ions induce greater precipitation than ferric ions, which is thought to be a result of its higher binding affinity, with Aβ aggregation enhanced to ~85 % [3]. In addition, Cu^2+ ^ions are bound at the low affinity site completely displacing the Zn^2+ ^ions in physiological acidosis [2]. Levels of copper, iron and zinc have been analyzed in the rims and cores of senile plaques of AD patients and concentrations were found to be highly elevated. Concentrations exhibited in the senile plaques cores were 22.7 ± 6.5, 52.4 ± 14.5 and 67.0 ± 13.0 μg/g for copper, iron and zinc respectively [4]. In addition serum copper levels have been shown to be markedly elevated in AD [5].

The catalytic effects of transition metals on the formation of reactive oxygen species are becoming evident as a major factor in AD. Aβ binds to Cu^2+ ^reducing it to Cu^+^. The Aβ-Cu^+ ^complex can then trap dioxygen and reduce it to hydrogen peroxide (H_2_O_2_). One study demonstrated that 1 μM Cu^2+ ^when bound to Aβ could catalytically generate 10 μM H_2_O_2_, indicating continual redox cycling [6]. In conditions where this reaction is not favorable, biological reducing agents such as vitamin C have been shown to enhance this reaction [7]. The generation of H_2_O_2 _has been shown to be neurotoxic, which is considered to be a result of its reduction to the hydroxyl radical via Fenton chemistry. Cupric ion binding is dependent on histidine and tyrosine amino acid residues. Upon generation of hydroxyl radicals localized to the Cu^2+ ^ion, the tyrosine residues can cross-link to form dityrosine. This further stabilizes the Aβ plaques conferring resistance to proteolysis [8]. Further studies are required to establish if Aβ bound to Cu^2+ ^and Zn^2+ ^exhibits significant anti-oxidant enzyme activities (superoxide dismutase and catalase activities) in vivo, as many researchers have likened Aβ to the native CuZn superoxide dismutase enzyme in both its conformation and its metal binding affinities [9].

Magnetite and maghemite deposits have been found in brains of patients suffering from AD and studies are underway to correlate enhanced levels with disease progression [10]. However, magnetite detection is only possible after the onset of AD. A comprehensive understanding of the levels and nature of redox-active ferric ions is warranted to assess the mechanisms of magnetite deposition. AD brains exhibit increased levels of oxidatively modified nucleic acids, specifically RNA in vulnerable neurons [11]. Haem oxygenase-1 (HO-1), an enzyme which catalyses the breakdown of haem to biliverdin with release of iron and carbon monoxide, is over-expressed in the brains of AD patients. So it could be postulated that this would be one source of redox-active iron. Immunostaining of neurons demonstrated that HO-1 expression was co-localized to the neurofibrillary tangles [12]. However oxidative damage to nucleic acids was reduced in neurons exhibiting neurofibrillary tangles demonstrating an antioxidant mechanism for HO-1 and an involvement in tau proteins expression. Further studies on the oxidative modifications of RNA in AD demonstrated it to be localized to iron rich lysosomes called lipofuscins originating from the mitochondria. This indicates a disruption in mitochondrial function leading to accumulation of redox-active iron in the neuronal cytoplasm [13] and subsequent reactive oxygen species generation.

### Therapeutic Implications

Oxidative stress is one of the key mechanisms of neurotoxicity in AD, and studies have shown that Aβ neurotoxicity can be partially or completely attenuated by the antioxidant enzyme catalase [6,7]. Plasma levels of antioxidants including vitamin E, uric acid and vitamin C are depleted in AD patients [14]. Therefore, many people have proposed the supplementation of antioxidants such as vitamin E and vitamin C. Several studies have reported that increased intake either through diet or supplementation results in reduced incidence of AD [15-17]. However, other studies do not report such success [18]. In fact, some studies have shown that vitamin C which can also act as a pro-oxidant can also induce neuronal oxidative stress via its interaction with metal ions [19].

A long-term imbalance in metal ion homeostasis is rarely treatable by alterations in diet. Therefore, research is focusing on the development of new methods for therapeutic intervention, specifically chelators to remove the deleterious metal ions, with concomitant dissolution of the Aβ plaques. *In vitro *studies on the aggregation of Aβ induced by metal ions, showed that this was completely reversible by metal ion chelation [3]. As a result of these observations new chelators are being developed as novel therapies for AD, however many are restricted by their poor target specificity. Clioquinol a Cu/Zn chelator was tested in APP2576 transgenic mice and appeared to selectively bind the Aβ-metal complex resulting in dissolution of Aβ plaques, reducing brain Aβ by 49 % [20]. No neurotoxicity was exhibited. Clioquinol subsequently was tested in a clinical trial with 36 AD patients. It affected a rise in plasma concentrations of zinc, and also decreased plasma concentrations of Aβ 1–42 [21]. However more studies are required to determine its importance in treating AD. Many other metal ion chelators are currently under investigation for treatment of AD [22-25].

We have developed a series of metal ion chelators for both the study and treatment of metal deposition disorders. These include the development of the first-ever, reactive oxygen and nitrogen species probe for the simultaneous detection of multiple RONS and the role of redox-active metal ions in their generation and suppression [26]. Alteration of the basic structure of these probes affords anti-oxidant enzyme mimics [27]. In addition the chelator EGTA, which has previously been shown to dissolve Aβ plaques [28] was also demonstrated to have substantial superoxide dismutase activity when complexed to redox-active metal ions [29]. Thus, addition of a chelator that generates a superoxide dismutase or catalase mimic could 'dissolve' the magnetite deposits and Aβ plaques and negate oxidative damage arising from them.

## Conclusion

Metal specific chelators afford a major opportunity for the control of metal ion mediated pathogeneses, which is not currently provided by nutraceuticals. In particular, administration of "prodrug" chelators that mimic anti-oxidant enzymes with high specificity for Cu^2+ ^and Fe^3+ ^ions will: i) counteract oxidative stress, ii) help to control metal ion deposition and iii) dissolve Aβ plaques and, maghemite and magnetite residues.

## Competing interests

The author(s) declare that they have no competing interests.

## Authors' contributions

The authors contributed equally to this work.
